# A fairness framework for natural language processing in substance use disorders and overdose

**DOI:** 10.1371/journal.pdig.0001480

**Published:** 2026-06-05

**Authors:** David T. Zhu, Suhanee Mitragotri, Suzanne Tamang

**Affiliations:** 1 Medical‌‌ Scientist Training Program, School of Medicine, Virginia Commonwealth University, Richmond, Virginia, United States of America; 2 Harvard College, Harvard University, Cambridge, Massachusetts, United States of America; 3 Department of Medicine, Stanford University School of Medicine, Stanford, California, United States of America; 4 Department of Veterans Affairs, Program Evaluation Resource Center, Office of Mental Health and Suicide Prevention, Menlo Park, California, United States of America; University of Toronto - St George Campus: University of Toronto, CANADA

The overdose crisis claimed nearly 70,000 lives in the 12-month period ending December 2025 [1]. Researchers, clinicians, and public health agencies are increasingly interested in machine learning (ML) approaches, such as natural language processing (NLP), to identify patients at risk, extract timely clinical and toxicological information, and support targeted interventions [2]. These NLP tools are commonly implemented using deep learning architectures such as transformer-based models, recurrent neural networks (RNNs), and convolutional neural networks (CNNs), which are well-suited for modeling sequential and unstructured text data from sources such as clinical notes and social media posts [3]. In particular, transformer-based models are now widely used in modern NLP because of their ability to capture long-range contextual dependencies at scale, making them a common foundation for downstream training and fairness interventions.

Despite their promise, these tools also carry substantial risks. Without deliberate safeguards, NLP models may reinforce the same structural inequities that have long marginalized people who use drugs (PWUD), particularly if fairness is not prioritized in their design and evaluation [4]. “Fairness” entails *developing and applying algorithms that perform equitably across racial, socioeconomic, and geographic subgroups, while accounting for the historical and structural biases embedded in health and social data*. In this Opinion, we propose a conceptual framework for developing and deploying fairness-centered NLP applications in the overdose crisis. Specifically, we advance three pillars: bias detection and mitigation, equity-centered design, and community engagement. This framework builds on established ML fairness approaches, including equal opportunity and demographic parity, while extending fairness beyond model performance to address the structural inequities affecting patients with substance use disorders.

Firstly, NLP models must be explicitly evaluated in the populations most affected by the overdose crisis. Although Black and American Indian/Alaska Native populations experience disproportionately high overdose mortality rates [5], assessments of model bias or subgroup-specific performance in these groups remain uncommon. In one study, a NLP model trained to detect opioid misuse from clinical notes demonstrated an absolute false negative rate of 32% among Black patients, nearly twice the rate observed among White patients [6]. Missed cases among Black patients were associated with worse clinical outcomes, including higher risks of hospitalization and death [6]. If left unaddressed, such disparities may delay timely interventions for patients already facing structural barriers to care and further erode trust in health systems.

Even when models appear fair on average, uneven performance across subgroups undermines the principle of *equal opportunity fairness*, which holds that individuals at comparable risk should be equally likely to be flagged for intervention [7]. These inequities often remain hidden unless performance is explicitly disaggregated by race and ethnicity, socioeconomic status, or geography. Techniques such as subgroup-specific threshold calibration, which adjusts decision thresholds separately across groups to equalize error rates, offer one potential pathway to mitigate these gaps without sacrificing overall accuracy [6]. It should be noted, however, that key protected attributes such as race and ethnicity are often not reliably or consistently documented across electronic health record systems, complicating fairness evaluation and limiting the feasibility of subgroup-specific analyses. Future work should therefore explore complementary approaches to fairness assessment in settings with incomplete or misclassified demographic data.

Secondly, in considering equity-centered design, we must recognize that bias is often embedded in training data itself. Such bias may arise from multiple sources, including *representational bias* due to systematic underrepresentation of certain populations, *measurement bias* from differential documentation or coding practices, and *historical bias* reflecting entrenched structural racism within healthcare systems. Clinical documentation, a common input for NLP models, can contain stigmatizing or stereotypical language [8]. For example, Black patients are more frequently labeled as “noncompliant” or “agitated,” terms that reflect biased provider perception rather than objective clinical differences [8]. When models are trained naïvely on such data, they may learn to associate such descriptors with overdose risk because they reflect racialized stereotypes embedded in the data.

These concerns extend beyond clinical notes to other data sources increasingly used in overdose surveillance and prediction, including social media posts, toxicology reports, and emergency medical services narratives, all of which are influenced by social context and institutional practices. Similar risks apply to other medically underserved populations with substance use disorders, including rural populations, justice-involved individuals, people experiencing homelessness, and those with intersecting marginalized identities. Rather than treating such data as neutral sources of information, developers must interrogate what the model is learning and why.

Counterfactual data augmentation (CDA) offers one promising strategy to address these challenges. CDA improves fairness by training models to discount demographic signals that are not directly linked to risk [9]. It does so by generating matched inputs that differ only in protected attributes, such as race, gender, or dialect, while holding clinically-relevant content constant. For example, if a model assigns a higher risk score to “Black male, noncompliant” than to an otherwise identical note reading “White male, noncompliant,” CDA penalizes that discrepancy, while preserving differences driven by clinically meaningful descriptors. In this way, CDA encourages models to focus on clinically justified features rather than socially coded ones.

In a foundational sentiment classification study, Kaushik et al. demonstrated that models trained on counterfactual examples were significantly more robust to spurious correlations [9]. This principle is directly relevant to overdose-related NLP. By incorporating carefully designed “what-if” examples drawn from clinical notes or social media, models can learn to distinguish meaningful clinical signals from bias. When implemented thoughtfully, CDA strengthens both equity and validity by helping models learn what they should and unlearn what they should not.

Although CDA is model-agnostic and applicable across architectures, including transformers, RNNs, and CNNs, it can be computationally intensive and difficult to scale for large clinical datasets. Moreover, additional studies and reviews are needed to evaluate the effectiveness of CDA and other sources of algorithmic bias in clinical NLP and overdose prediction contexts. Such work must grapple with inherent tradeoffs among fairness objectives; for example, improvements in equal opportunity may come at the expense of demographic parity, necessitating explicit, context-specific decisions and further research to characterize these tradeoffs during model evaluation and deployment.

Thirdly, artificial intelligence tools intended to help communities should be developed *with* them, not just *for* them. Despite increasing recognition of this need, fewer than 0.2% of studies on artificial intelligence in healthcare broadly report any form of community involvement [10]. This lack of inclusion not only undermines trust but weakens technical performance by overlooking lived experiences that are essential to understanding evolving drug trends and often fails to adequately address community needs to meet people where they are at.

Community engagement is particularly salient for NLP applications related to xylazine, a veterinary sedative increasingly detected in the illicit drug supply and associated with severe skin wounds and prolonged sedation [5]. NLP-based surveillance of xylazine highlights the value of community-based participatory design approaches, including community forums, participatory annotation, and compensated collaboration with PWUD. In one study, researchers applied Reddit-based NLP to identify emerging discourse, then consulted with PWUD to validate and contextualize the findings [11]. Future efforts could also involve PWUD during *study conceptualization* (e.g., identifying relevant outcomes, data sources, and terminology), *model deployment* (e.g., determining appropriate use cases and safeguards to minimize harm), and *post-deployment monitoring and evaluation* (e.g., assessing changes in drug use over time, misclassification, or unintended consequences).

Within the context of a rapidly evolving overdose crisis, such engagement provides essential domain knowledge to refine model inputs and outputs. Community participants could help identify colloquial terminology, such as “tranq” or *anestesia de caballo* (“horse anesthesia”), that may otherwise elude traditional keyword-based detection methods in electronic health record data. They can also shed light on emerging patterns of polysubstance use, including combinations of xylazine with opioids and stimulants in “speedball” formulations that are increasingly common [5]. Integrating this experiential knowledge into NLP development improves contextual sensitivity and ensures alignment with lived realities of substance use.

Despite these benefits, community-engaged NLP research has important limitations that warrant careful consideration. These include ethical concerns related to privacy, consent, and the risk of reinforcing stigma or surveillance-related harms. Implementation may also pose feasibility challenges for smaller health systems and under-resourced public health settings, emphasizing the need for scalable, context-appropriate engagement and governance models. Clear compensation frameworks, data governance agreements, and explicit boundaries around how model outputs are used are essential to ensure that participation is not extractive and that resulting tools benefit, rather than harm, the communities most affected. When implemented thoughtfully, however, community engagement can strengthen the ethical development and deployment of NLP systems addressing xylazine and the broader overdose crisis.

NLP presents a powerful tool to strengthen epidemiological surveillance and public health interventions in the face of a rapidly evolving overdose crisis. As these technologies gain traction, researchers and public health systems face a critical opportunity to advance equity by embedding fairness throughout the entire pipeline, from model development and validation to procurement, deployment, and downstream use.

For researchers and NLP developers, this may involve treating bias detection and mitigation, equity-centered design, and community engagement as core design objectives rather than a post hoc consideration. For clinicians and public health agencies, it may include integrating fairness audits into implementation decisions, monitoring performance across populations over time, and seeking ongoing feedback from clinical and public health professionals. Sustaining these efforts will require long-term institutional and public investment in data infrastructure, interdisciplinary expertise, and community partnerships, rather than one-time pilot funding. Ultimately, a fairness-centered approach to NLP in the overdose crisis demands coordination across research, practice, and policy to ensure that these technologies improve surveillance and prediction in ways that are equitable, accountable, and responsive to the communities most affected by substance use disorders and overdose.
